# Role of TNF-A, IL-6 and IL-4 with the susceptibility to chronic periodontitis in North Indian population: a multi-analytic approach

**DOI:** 10.1186/1755-8166-7-S1-P72

**Published:** 2014-01-21

**Authors:** Garima Prakash, Sadhna Ajay, KK Gupta, Balraj Mittal

**Affiliations:** 1 Dept. of Genetics, SGPGIMS, Lucknow, India; 2 Dept. of Periodontics, SPPIDMS, Lucknow, India

## Background

Periodontitis is a chronic inflammatory disease causing destruction of tooth supporting tissue and loss of teeth. Inter-individual variation in genetic factors and one or more variations in genes could play critical role in susceptibility to Chronic Periodontitis and its pathogenesis. Therefore, in present study, we aimed to perform combined risk genotype analysis and Multifactor dimensionality reduction (MDR) to identify combination of alleles in modifying the risk of chronic periodontitis.

## Materials and methods

A total of clinically diagnosed 200 periodontitis cases and 200 age and gender matched controls were genotyped for TNF-α, IL-6 and IL-4 gene polymorphisms using ARMS PCR and PCR-RFLP methods. Multi-analytic approach (combine risk genotype analysis and MDR) were used to find out the combination of allele contributing to risk of chronic periodontitis. All statistical analyses were performed using SPSS ver.15.0 and MDR analysis by MDR 2.0 Beta 8.4.

## Results

Single locus analysis showed association of TNFα-308GA (rs1800629) with risk of CP whereas IL-6-174GC (rs1800795) and IL-4-590CT (rs224325) polymorphisms did not show any significant differences. However, combined risk genotypes of all three polymorphism depict the increased risk of CP (P_trend =_0.001) where MDR analysis revealed TNF-α-308GA and IL-6-174GC as a best polymorphic model for the risk of CP. When subjects were stratified on the basis of gender, only TNFα-308GA polymorphism showed association with risk of CP in North Indian population.

## Conclusion

TNF- α -308G>A polymorphism is associated with chronic periodontitis. However, multi-analytical approach suggests TNF-α-308GA and IL-6-174GC genetic variants interact to confer significant risk for chronic periodontitis.

